# Effectiveness of Multiple Blood-Cleansing Interventions in Sepsis, Characterized in Rats

**DOI:** 10.1038/srep24719

**Published:** 2016-04-21

**Authors:** Ivan Stojkovic, Mohamed Ghalwash, Xi Hang Cao, Zoran Obradovic

**Affiliations:** 1 Center for Data Analytics and Biomedical Informatics, College of Science and Technology, Temple University, 19122, Philadelphia, PA, USA; 2 Signals and Systems Department, School of Electrical Engineering, University of Belgrade, 11120, Belgrade, Serbia; 3 Mathematics Department, Faculty of Science, Ain Shams University, 11566, Cairo, Egypt

## Abstract

Sepsis is a serious, life-threatening condition that presents a growing problem in medicine, but there is still no satisfying solution for treating it. Several blood cleansing approaches recently gained attention as promising interventions that target the main site of problem development–the blood. The focus of this study is an evaluation of the theoretical effectiveness of hemoadsorption therapy and pathogen reduction therapy. This is evaluated using the mathematical model of Murine sepsis, and the results of over 2,200 configurations of single and multiple intervention therapies simulated on 5,000 virtual subjects suggest the advantage of pathogen reduction over hemoadsorption therapy. However, a combination of two approaches is found to take advantage of their complementary effects and outperform either therapy alone. The conducted computational experiments provide unprecedented evidence that the combination of two therapies synergistically enhances the positive effects beyond the simple superposition of the benefits of two approaches. Such a characteristic could have a profound influence on the way sepsis treatment is conducted.

Sepsis is a potentially life-threatening complication of pathogen infection that triggers the systemic inflammatory response^1^. Such systemic inflammation can initiate a cascade of processes that can lead to multiple organ dysfunction syndrome and even death^2^. Furthermore, sepsis afflicts a large population^3^ and is often diagnosed too late, which can result in a mortality rate as high as 30–50% in the case of septic shock^4^. In fact, sepsis has been recognized as one of the main causes of in-hospital deaths in the United States^5^, with more than 750,000 cases annually^6^, and it contributes to 1 in every 2 to 3 deaths^7^. The financial side of the problem should also be taken into account, since sepsis is a very expensive condition with over $20 billion of hospital costs in 2011 only in the United States^8^. Therefore, sepsis treatment is a highly important problem, and the necessity of finding an effective solution will only grow, as the incidence of sepsis is rising^9^.

Various approaches for treatment of sepsis were proposed and tested over the last few decades^10^,^11^, but most showed a limited ability to significantly improve the outcome. The single approved anti-sepsis drug therapy was withdrawn from global markets in Fall 2011, following the failure of its worldwide trial to demonstrate improved patient outcome^12^. Nevertheless, the state-of-the-art and the most effective treatment remains application of antimicrobial drugs^1^. It is acknowledged that antibiotics have limitations, including inefficiency in treating pathogens other than bacteria. Broad spectrum solutions are not as effective as specialized ones, but the latter require identification of the pathogen, which is too time consuming given the rapid course of sepsis. In addition, inappropriateness of empirical antibiotic therapy can contribute to high level of mortality^13^ and frequent use and abuse of antibiotic drugs leads to the evolution of more antibiotic resistant strains that will further reduce their efficacy^14^.

Although sepsis complications and severity depend on the way it affects organs, sepsis is initiated by spillover of pathogens into blood, which is the medium that allows the pathogen to spread throughout the organism and in which systemic inflammation takes place. Use of blood purification showed promising results in other critical illness conditions, such as acute kidney failure^15^,^16^. Therefore, blood cleansing approaches gained attention as possible and potentially effective therapies for septic subjects^17^,^18^,^19^. In such interventions, blood is purified by an extracorporeal device that removes harmful particles, leading the subject towards a healthy state. Inflammation reducing extracorporeal blood purification therapy, known as hemoadsorption (HA), was proposed and found to be beneficial in animal models of sepsis^20^,^21^. More recently, a blood-cleansing device for sepsis therapy inspired by the spleen was proposed^22^. This device can continuously remove pathogens and toxins from blood by using magnetic “nanobeads” that capture a broad range of pathogens and toxins without the need to first identify the infectious agent. The application of a biospleen approach in *animal models* of sepsis confirmed the ability of the approach to greatly reduce the pathogen levels in the blood^22^. In this article we will refer to this biospleen technique as pathogen reduction (PR) therapy.

The initial success of the mentioned blood cleansing techniques suggested their potential and encouraged further research in that direction^23^. This also motivated us to investigate and assess the characteristics of two proposed blood cleansing therapeutic approaches^22^,^24^ using a coarse-grained phenomenological model of the acute inflammatory response in cecal ligation and puncture (CLP)-induced sepsis in rats.

Animal models of sepsis remain an essential tool in understanding sepsis as well as in the development of novel therapeutic solutions^25^, in-spite of failure to validate promising therapies in clinical trials^12^ and findings that question the utility of animal models to study human sepsis^26^. Animal sepsis research provides invaluable insights into underlying mechanisms, given that it allows extensive experiments while controlling many important conditions from cause^27^ to phenotype^28^ and even gene expression manipulation^29^,^30^. After all, ethical guidelines such as the Nuremberg Code and Declaration of Helsinki (although not legally binding), require research involving Humans to be based on knowledge obtained from animal experiments^31^. The CLP model of sepsis is often referred to as the golden standard^32^ in sepsis research due to its ability to reproduce the dynamics observed in clinical sepsis in human^33^, although, as any animal model, it has its limitations regarding the relevance to human sepsis. A major limitation of the CLP rat model is the size of the animal and particularly its cardiovascular system, where larger animals, such as pigs, would have more relevance in that aspect. In fact, thorough evaluation of any promising treatment for sepsis should involve assessment using a series of animal models of increasing complexity^32^.

In this article, a published mathematical model of animal sepsis is utilized to interrogate the *theoretical* effectiveness of the blood cleansing treatments. The criteria of effectiveness are defined and adopted to measure the utility of interventions. The mathematical model originally proposed in^24^ is extended to incorporate the pathogen reduction therapeutic effects^22^, and thoroughly evaluate various modalities of hemoadsorption and pathogen reduction therapies. Under the null hypothesis that there is no difference between the two therapeutic approaches, we have performed a number of simulations under the equivalent conditions and summarized the outcomes. Evidence obtained by comparing over 2,200 configurations of hemoadsorption and pathogen reduction therapies and combination thereof on 5,000 subjects shows that blood cleansing therapies are the most effective when applied early and for a longer time. It is also found that pathogen reduction therapy is more effective than hemoadsorption therapy. However, it is observed that neither of the approaches alone is able to rescue all subjects, and each of them only *partially* solves the problem. Subsequently, we hypothesized that removing *both* the inflammatory mediators and pathogen cells would have more thorough effects than either therapy alone. We have tested another null hypothesis that a combination is not better than a single approach by means of three different methods: by directly comparing numbers of rescued subjects using all tree therapeutic options, using the optimization techniques to find the optimal therapy configuration, and by characterizing synergistic interactions using isobole analysis. The obtained results provide evidence that the combination of the two therapies is more effective than the application of either therapy alone. This is the first study suggesting that the combination of different interventions could provide substantial improvement of therapeutic effects over a single intervention. Such insights can bring more attention to the potential of combining multiple approaches as a more effective way to fight sepsis. Consequently, research efforts might be directed to find the optimal application setup of existing therapeutic approaches rather than exploring only new revolutionary approaches for sepsis treatment.

## Methods

Recently, a mathematical model of acute inflammatory response in sepsis was developed in order to better understand the pathophysiologic mechanisms of sepsis^24^. The model was built using heuristic knowledge about the mechanisms underlying the acute inflammation process in response to infection. In order to produce a more realistic model, the probability distributions of physiological parameters of the model were constrained based on *real experimental measurements* from septic rats, so that the model is able to reproduce observed patterns, while also representing a heterogeneous population, data uncertainty and other unexplained sources of variability. The objective of the model was to characterize the range of possible physiologic responses in a population, gain insight into the pathophysiology of severe sepsis, and generate testable hypotheses that guide future experiments. This has made the model well suited for incorporating other interventions. Consequently, we have utilized the model as the basis of our research to assess the effectiveness of multiple blood cleansing therapies. It allows us to perform *extensive* empirical evaluation through computational simulation to observe plausible effects of the blood filtration in sepsis.

### The mathematical model of sepsis

Here we will briefly introduce the mathematical model of CLP sepsis in rats and describe its main aspects. For the most complete and thorough description of the model, readers are directed to the Supplementary Information (SI) Appendix and the original article^24^.

The model^24^ is comprised of 19 states corresponding to important physiological variables that govern sepsis dynamics. Some of these variables are observable, such as concentrations of different cytokines, cells like neutrophils, and pathogen levels. The other variables are conceptual, such as total tissue damage and lumped state of pro-inflammation or anti-inflammation. These variables influence each others’ progression and that dependency is described through a set of 18 ordinary differential equations (ODEs), which are based on domain knowledge, accompanied with one external signal representing an initial pathogen challenge that triggers the response. This set of ODEs together governs the evolution of all states, resulting in different temporal patterns according to the values of the free parameters. The model is calibrated using real measurements gathered from the experiments on CLP septic rats^24^. The values of the free parameters are chosen such that the generated model trajectory closely follows experimentally observed temporal patterns.

### Process of generating virtual subjects

The model is used to generate a population of solutions, referred to as *virtual subjects*, that have a trajectory similar to the experimental observation (SI Appendix Fig. S2). The virtual subjects can depict different phenotypes (eg. survival versus non-survival) through appropriate values of parameters and states’ initial conditions used to account for the observed diversity in real experimental subjects. We have used the model to generate a population of virtual subjects in which the parameters were initialized and then the subject states were evolved according to the ODEs through the simulation of 200 time units (hours). At the end of the simulation the subject belongs to the non-survival group if pathogen level or pro-inflammation level are above certain thresholds^24^. Otherwise, the subject belongs to the surviving group.

For the purpose of conducting computational experiments we have generated 5,000 virtual subjects that exhibit the properties of non-surviving subjects, using the code provided by the respective authors^34^. Parameters of those subjects were sampled from Monte Carlo Markov Chain obtained using the Metropolis-Hastings method^35^. As shown in SI Appendix Fig. S2, the generated virtual subjects follow similar trajectories of sepsis dynamics as the experimental observations of eight cytokines measured in CLP rats that did not survive one week after the induction of sepsis^24^.

### Modeling hemoadsorption and pathogen reduction therapies

The model^24^ already incorporates the theoretical mechanism behind hemoadsorption therapy. As modeled, hemoadsorption therapy removes the inflammatory mediators (pro- and anti-inflammation levels as well as activated neutrophil levels) as long as the therapy is active. In addition, we have extended the mathematical model to include the theoretical mechanism of pathogen reduction therapy that removes the pathogen from the blood as long as the therapy is active^22^. The patient states’ evolution can be modulated by applying the blood purification device (hemoadsorption or pathogen reduction) for a certain period of time and then the outcome (subject state) at the 200^th^ hour can be assessed according to the *survival criterion*. The survival criterion classifies the subject as survived if the pathogen levels are below 10^5^ and the pro-inflammation level is below 0.5, and non-survived otherwise^24^. The details of the overall mathematical model with therapeutic interventions are explained in the SI Appendix.

### Evaluating the efficacy

Regardless of the type of blood cleansing, the question of how to apply blood cleansing therapy appropriately (to achieve best therapeutic effects) is very important. Assuming the discrete nature of the approach (blood is either filtered or not, because controlling the degree of filtration is still not an option), the question of when to apply it and for how long still remains. It seems like a two part problem, but in fact it may be much more diverse, since the blood filtration may be applied in several separate time intervals over the course of disease treatment. Although multiple interrupted filtrations are valid and maybe even potentially beneficial^36^, in this study we will assume only one interval of continuous application per approach (pathogen reduction or hemoadsorption) for presentation and computational convenience. Therefore, for a single blood cleansing approach, the problem is to decide *starting* time (when to apply) and *duration of* (how long to apply) the therapy. In the case of a combination of the two approaches, there will be separate pairs of starting and duration times for each of the two blood cleansing approaches. Hereafter, we use the term *“configuration”* of the therapy to refer to a particular combination of starting and duration times. Using the model, we have extensively evaluated different configuration setups of therapies to characterize their efficacy.

Here, we will explicitly define *the efficacy* criteria by which one may compare different therapeutic approaches. For a particular configuration (start and duration) of a particular blood cleansing approach (pathogen reduction or hemoadsorption) and a particular virtual subject, it can be said that therapy is effective if the subject is classified as rescued after receiving it, and not effective otherwise. Such a binary measure of efficacy can be summarized over a number of simulations in various ways to characterize different therapeutic settings in population subsets. For example, the efficacy of a configuration of a therapy over population of virtual subjects can be measured as a percentage of effective applications. We have exploited the described notion of efficacy in our analysis, however, it is not the only one. Configurations of therapies evaluated in some subjects may also be assigned (and compared by) continuous label values of efficacy based on (for example) how much they have reduced pathogen or inflammation levels. Such a measure of efficacy was employed in *isobole* analysis, where we were interested in the ability of a therapy to decrease inflammatory mediator levels. The formulation of efficacy may be generalized even further by taking into account both the outcome class of treated subjects and pathogen level reduction, as well as the length of therapy application and similar characteristics. We have defined this more general criterion and used it in order to find the optimal therapeutic setting, all of which will be described in details in the Results section.

The efficacy of proposed therapeutic solutions was computationally evaluated by three means:

- Forward evaluation of virtual subjects on a grid of possible therapeutic configuration, and summarization of obtained outcomes.
- Therapy applications in virtual subjects were simulated for a representative subset of possible configurations that were equally distributed on a two-dimensional grid of starting and duration times. The overall effectiveness of therapeutic approaches was measured using different aggregation schemes of particular applications and a binary definition of particular configuration effects (successful or not).
- (Inverse) evaluation of virtual subjects by the optimization procedure to find the best therapeutic configuration according to suitably defined criteria.
- Using the combined criterion of effectiveness along with the constraints on application times as a formulation of the optimization problem, the optimal therapy configuration for each virtual subject was obtained by solving posed nonlinear constrained optimization using a global metaheuristic search^37^.
- Isobole analysis to assess the interactions of the two therapeutic approaches.

This common tool in drug interactions analysis^38^ was used to access intervention interactions through their inflammation reduction potential (continuous definition of effectiveness).

## Results

In this section, we present the results obtained using the methodology briefly described in the previous section to test the two main hypotheses of this study. First hypothesis, that there is a difference between the two blood cleaning approaches, and the results obtained from testing it, were used as a motivation for stating and investigating the second one. The second and central hypothesis of this work is that the combination of therapies is more effective than each one individually. While the main results are covered in this section, additional results may be found in the SI Appendix, and source codes used in generating outlined results are available online^39^.

### Hemoadsorption therapy evaluation

We have evaluated the effectiveness of hemoadsorption therapy to show both an estimate of subjects who could benefit from the therapy, and the configurations that lead to more effective therapy application in terms of starting time and duration of the therapy. We have generated 5,000 non-survival virtual subjects and *extensively* evaluated the hemoadsorption therapy for 744 configurations over a wide range of starting and duration times of therapy application. We have varied the starting time bi-hourly from 0 to 60 hours after the induction, and the duration time hourly from 1–24 hours. Obtained results for all subjects can be seen as a 3-D array with dimensions *start* × *duration* × *subjects* (31 × 24 × 5000), where each entry (*i*, *j*, *k*) is either 1 if the subject *k* survives when receiving *j* hours therapy starting at time *i*, and zero otherwise. Deploying different aggregation schemes on the 3-D array revealed interesting statistical results shown in the next paragraphs.

**Figure 1:**
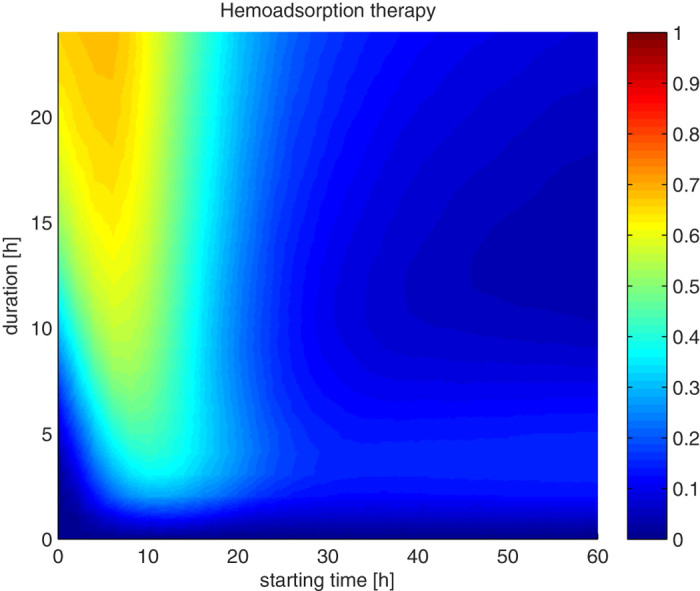
The efficacy of 744 configurations of HA therapies on 5,000 virtual subjects over a range of starting times and duration. Each pixel color represents the rate of subjects rescued using that configuration of starting time and duration. Warmer tones represent higher, and the colder tones lower survival rates (1 corresponds to 100% rescue). Earlier and longer treatments tend to save more subjects.

We have converted the 3-D array into a 2-D array by aggregating over 5,000 subjects. The resulting normalized 2-D array is shown in Fig. 1 where the x-axis represent the *starting time* and the y-axis represent the *duration* of the therapy. Each pixel in the figure shows the percentage of subjects rescued when using that therapy configuration. Different colors correspond to different percentages of rescued subjects. Warmer colored areas indicate that the corresponding therapy can rescue more subjects than areas with colder tones.

As shown in Fig. 1, it can be seen that (1) earlier and longer applications of hemoadsorption therapy can save more subjects than delayed or shorter application of the therapy, and (2) after 20 hours from infection, the duration of the therapy does not significantly affect the efficacy of the intervention. There is an obvious trend that longer duration would result in more rescued subjects, at least in the earlier stages of sepsis. However, the use of extracorporeal blood filtration devices leads to increased risk of trombogenesis^40^ and other undesired side effects^41^. That is why it is commonly administered with the use of anticoagulants like heparin. Even so, longer applications of heparin increase risk of systemic anti-coagulation and hemorrhage and are sometimes contraindicated. In any case there is always a trade-off between benefits and risks^42^ and longer applications of blood filtration should be carefully administered. Therefore, we have decided to adopt 24 hours as the maximum allowed continuous administration of therapies.

In previous research on hemoadsorption therapy^24^ it is reported that application of 4 hours of hemoadsorption therapy starting at the 18^th^ hour after the induction is able to rescue about 18% of non-survival virtual subjects (black point in SI Appendix Fig. S3). In addition, a recent article^43^ presented results evaluated for three different durations (4-, 8-, 12-hour therapy applications) at different starting times as shown in SI Appendix Fig. S3. The results from the literature are consistent with our findings.

### Severity of infection

We have also characterized the severity of the infection under the assumption that if the condition is not severe, then most configurations would help the subject to survive. Otherwise, only a limited number of total configurations would be effective, and all other configurations would not be able to rescue the subject. To depict that, we have converted the 3-D array into a 1-D array by aggregating over all *starting times* and *duration times*. The resulting matrix indicates the number of successful applications of therapy for each virtual subject. The results are shown in SI Appendix Fig. S4.

For ease of presentation, we have categorized all subjects into five bins according to the percentage of successful treatments. For example, (0–10%) indicate the percentage of subjects who could be rescued by some particular *x* ∈ (0–10)% of configurations (e.g., 10% out of all 31 × 24 = 744 possible configurations). In other words, some subjects in that bin could be rescued by 1% of configurations, some other subjects could be rescued by 2% of configurations, and so on. Therefore, all subjects who could be rescued by some *x* ∈ (0–10)% of configurations are categorized in the bin (0–10)%.

It is found that about 12.3% of 5,000 virtual subjects could be saved by more than half of all tested configurations (less severe subjects) while 25% of subjects could not be saved by any combination of starting and duration times (very severe cases). It shows that a large fraction of virtual subjects, one in every four, could not benefit from hemoadsorption therapy. Therefore, removing only the inflammation mediators was not enough for these subjects to be rescued.

Next, we investigated whether the same 5,000 virtual subjects could be rescued by removing the pathogen cells directly using the pathogen reduction therapy.

### Pathogen reduction therapy evaluation

The other intervention that has been proposed recently for blood cleansing is pathogen reduction therapy, which is based on a biospleen approach^22^. This intervention is different from hemoadsorption therapy in the way that it directly removes pathogen cells from the infected blood, and not the inflammatory mediators. Similarly to hemoadsorption therapy, pathogen reduction results in decreased levels of inflammation, but as a consequence of pathogen reduction, and not by directly removing inflammatory mediators from circulation. The pathogen reduction therapy uses magnetic nanobeads coated with an engineered human opsonin–mannose-binding lectin, which are capable of attaching to a wide range of sepsis inducing pathogens, including fungi and viruses, which are later extracted from the blood in an extracorporeal device inspired by the human spleen. For one hour of blood filtration in a septic rat, pathogen reduction removes more than 90% of the pathogens^22^. It should be pointed out that experimental results^22^ are obtained using intraperitoneal injection of the pathogen, which is a different sepsis model than CLP. While live pathogen injection is a nonsurgical model, it has a fast onset of disease and is more of an endotoxicosis model, whereas CLP is a surgical, polimicrobial method with gradual development of disease and symptom patterns well aligned with clinical sepsis^44^. Since we relied only on the results regarding the rate of pathogen removal, the differences in the progression of disease between the two experimental models should not affect our model. However, since *in vivo* removal rates in^22^ are measured on a well determined bacterial strain of S. aureus and *E. coli*, while CLP results in polymicrobial bacteremia of nonstandard composition, we will assume that average removal performance on such a spectrum of pathogens corresponds well to that of S. aureus or *E. coli* (90%). That assumption seems to be well supported by *in vitro* results^22^ where rates of removal of anaerobic and aerobic cecal bacteria were 98 and 80%, respectively.

To test the efficacy of the pathogen reduction therapy, we have modified the mathematical model to include a term that controls the direct removal of pathogens. Details about the extended model are described in the SI Appendix. Simulations on the previously generated 5,000 virtual subjects are repeated under the same conditions as with hemoadsorption therapy but utilizing pathogen reduction therapeutic approach.

**Figure 2:**
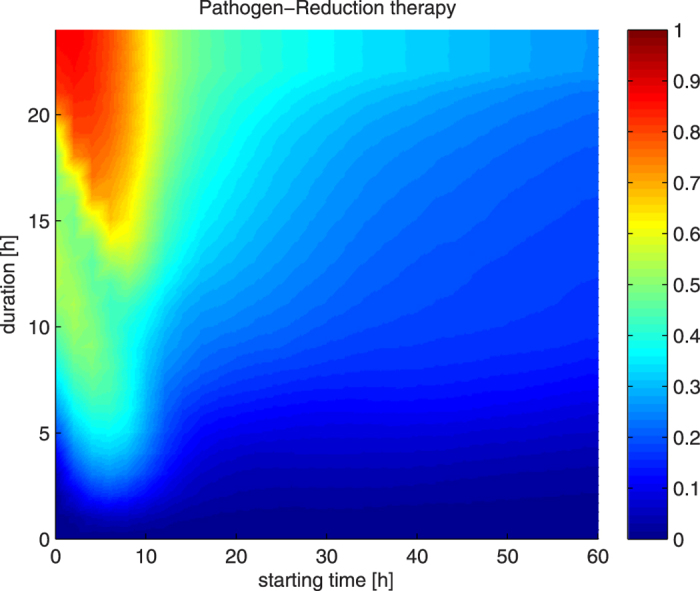
The efficacy of 744 configurations of PR therapies on 5,000 virtual subjects over a range of starting times and duration. Each pixel color represents the rate of subjects rescued using that configuration of starting time and duration. Warmer tones represent higher, and the colder tones lower survival rates (1 corresponds to 100% rescue). Earlier start and longer treatments tend to rescue more subjects.

Results of pathogen reduction therapy efficacy on 5,000 virtual subjects are shown in Fig. 2. The figure shows similar trends as in the case of hemoadsorption therapy: earlier and longer applications of pathogen reduction therapy are preferred. However, higher percentages of rescued subjects can be noticed with the use of pathogen reduction therapy, compared to the use of hemoadsorption therapy. For example, an application of 20 hours of hemoadsorption vs. pathogen reduction therapy starting 4 hours from infection saves 66.1% vs. 81.6% of 5,000 virtual subjects, respectively. Another difference is that even after 20 hours from the induction, longer pathogen reduction therapy applications can make a noticeable difference in the number of saved subjects, which is not the case with hemoadsorption therapy.

### Severity of infection

The results of testing the severity of the infection and the ability of pathogen reduction therapy to rescue these 5,000 virtual subjects are shown at Fig. S5 in SI Appendix. As presented in the figure, only one tenth of all subjects could not be rescued by any combination of pathogen reduction starting and duration time, compared to a quarter in the hemoadsorption therapy approach. Therefore, the pathogen reduction therapy was able to save more virtual subjects than hemoadsorption therapy. However, there is still a group of subjects who also could not be rescued by pathogen reduction therapy.

### Comparison between hemoadsorption and pathogen reduction therapies

We have compared the two different therapeutic approaches in terms of percentage of subjects that could (or could not) be saved by either therapy, respectively. The counts are summarized in Fig. S6 in SI Appendix. It shows that 66.6% of 5,000 virtual subjects (3330) can benefit from either of the two therapies if therapies are applied *appropriately*, while the number of subjects that could benefit *only* from pathogen reduction therapy (1177) is substantially larger than the number of subjects that could benefit *only* from hemoadsorption therapy (422). This aggregated group which benefits from exactly one approach (32% of subjects) is really affected by the appropriate choice of therapy. If treated by the opposite one, they wouldn’t have a chance to recover. In addition, there exists a group of 71 subjects who could not benefit from either of the therapies alone. This group of 71 subjects can be seen as the most severe cases and can be really valuable in assessing the effectiveness of the proposed combination of therapeutic approaches.

### Assessing the effects of hemoadsorption and pathogen reduction therapies using optimization

We test whether the subjects could have more benefit from the combination of hemoadsorption and pathogen reduction therapies. However, this experiment, if conducted in the same manner as in the single therapy cases, would be very computationally expensive because for each subject we need to test 24 × 31 × 24 ×31 = 553,536 configurations, which would take weeks of computation on contemporary machines. Therefore, we find evidence that the combination of therapies is more beneficial than a single therapy by formulating the problem as finding the optimal therapy in the combined setup. The optimization algorithm finds the best configuration according to some suitable optimality criterion that takes as parameters: *hemoadsorption starting time*, *hemoadsorption duration*, *pathogen reduction starting time*, and *pathogen reduction duration*. The details are described in the SI Appendix, but we can say that the criterion evaluates configurations based on how well and with how much effort (dose) the subject is rescued. In the case when the combination is no better than a single approach, the optimization algorithm chooses only one therapy, and the application duration of the other one will be zero. The obtained solution that minimizes the optimization function indicates when to turn on and how long to apply either therapy for each subject. We have solved this optimization problem for each of the 5,000 subjects. The distribution of the ten most informative states, pathogen, pro-inflammation levels, and eight cytokines’ concentrations in subjects without therapy and with prescribed optimal therapy, are depicted in Fig. S7 in SI Appendix. After the spiking in the states as a reaction to initial stimulus, a steady decrease towards the nominal levels can be observed when the optimal combination of pathogen reduction and hemoadsorption therapies is applied. Such a pattern carries the message of resolving the sepsis and returning to the healthy state. Most importantly, the values of pathogen and inflammation levels are decreasing substantially below the threshold for declaring the subject as a (non-)survivor.

Note that when the optimization algorithm finds that it would be optimal to apply both therapies, it does not mean that the subject could not be saved by a single therapy alone. It just means that according to our criteria, the intervention by both therapies minimizes the optimization function more than the intervention by either therapy alone. For example, let’s imagine that 12 hours of hemoadsorption could make a subject as healthy as applying 3 hours of pathogen reduction and 6 hours of hemoadsorption. In such a case, the optimality criterion would favor a combination because it can make a subject as healthy as a single therapy, but using shorter therapy. Similarly, for two therapies of the same total duration, the one that results in lower levels of pathogen and inflammation would be chosen by the optimization algorithm.

Table 1 shows the fractions of subjects that would have most benefit from each of the three therapies: hemoadsorption only, pathogen reduction only, or Combination. Obtained recommended therapies were able to rescue the targeted subjects except one subject (out of 5000 subjects). That subject requires longer total therapy duration than what we have allowed in our optimization setup (24 hours). As shown in the table, the majority of the subjects (65%) would most benefit from the combination of the two therapies, which means that, according to our optimization, the combination of the two therapies is more effective for those subjects than applying each therapy separately. This group of 65% of subjects who were rescued by the recommended combined therapy contains most of the previously incurable ones (70 out of 71 subjects) who could not be saved by *either* of the therapies individually (SI Appendix Fig. S6). This validates our hypothesis that, in some cases, removing only one of the causes of sepsis is not enough to treat the subjects and sometimes it is necessary to remove both the inflammatory mediators and pathogen cells from the circulation. This was indeed the case in those 70 subjects.

### The most effective combination of pathogen reduction and hemoadsorption therapies

We have shown that the optimized combination of therapies is more effective than individual therapy. However, it is not realistic to obtain in advance the best combination for treating a particular septic subject because that would require complete knowledge of both the subject’s individual characteristics, and the exact infection time. Although highly desirable, personalized treatments are really challenging in practice, and usually all subjects are treated with the same configuration. Therefore, we have explored which particular combination would rescue as many subjects as possible and would have the best chance for success on average.

**Figure 3:**
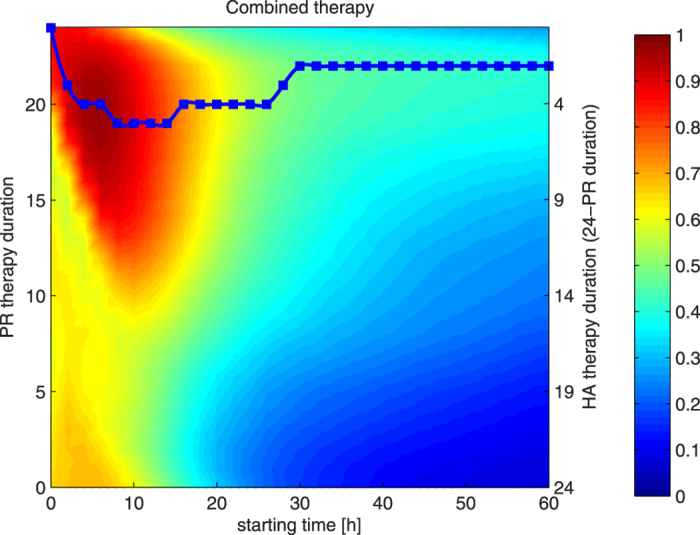
The efficacy of combined therapies on 5,000 virtual subjects when starting therapy within 60 hours of infection and limited the total therapy to 24 hours. Each pixel color represents the rate of subjects rescued using that configuration of starting time and duration. Warmer tones represent higher, and the colder tones lower survival rates (1 corresponds to 100% rescued rate). The blue line represents the maximum percentage of rescued subjects. Combined therapies where pathogen reduction therapy is much longer than hemoadsorption were the most effective.

There is an obvious trend in Figs 1 and 2 that the longer the therapy the more rescued subjects, particularly in the early stages of sepsis. We assumed that such a pattern would also be present in the combination of therapies. So, in order to find a single configuration that has the best chances to rescue a subject, we have focused on boundary cases with maximum allowed total duration, since we expect that this should be the most effective configuration. We have fixed the total duration (aggregated time) of both therapies to 24 hours and set both starting at the same time. To conduct such an experiment, we have varied the duration of pathogen reduction therapy hourly from 0 to 24 hours and conversely hemoadsorption therapy from 24 hours down to 0 hours of application so that the total duration for both of them would be exactly 24 hours. The starting time was changed bi-hourly from induction time up to 60 hours post-induction. The results are shown in Fig. 3.

The x-axis in Fig. 3 represents the starting time for both therapies, while pathogen reduction (hemoadsorption) therapy is applied for a duration represented by left (right) y-axis, respectively. One can notice that the horizontal line at the 24^th^ hour of pathogen reduction application in Fig. 3 (top horizontal line) corresponds to the horizontal line at the 24^th^ hour in Fig. 2 (top horizontal line) and the horizontal line at the 24^th^ hour of hemoadsorption application in Fig. 3 (bottom horizontal line) corresponds to the horizontal line at the 24^th^ hour in Fig. 1 (top horizontal line). This figure confirms the initial finding we discussed before. Figure 3 shows that (1) the maximal performances lies in between these boundary horizontal lines as marked with the blue line, advocating that the combination of approaches is indeed better than any of the approaches alone, and (2) the pattern that early application of the combined therapy is more effective is again visible here. The very best configuration, obtained with conducted simulations, requires application of 20 hours of pathogen reduction and 4 hours of hemoadsorption starting from the 6^th^ hour after induction, and is able to rescue 97% of subjects. We can see that the starting time is also crucial. If we follow the performance of the blue line we see that it is going to decrease eventually. This blue line could be taken as some sort of prescription (recipe) for conducting most appropriate treatment, if an estimate of induction time can be made. But even if it is not the case, the blue line shows that pathogen reduction should be applied considerably longer than hemoadsorption; with about 21 hours of pathogen reduction and 3 hours of hemoadsorption we would not be far away from the best possible configuration at any time.

**Figure 4:**
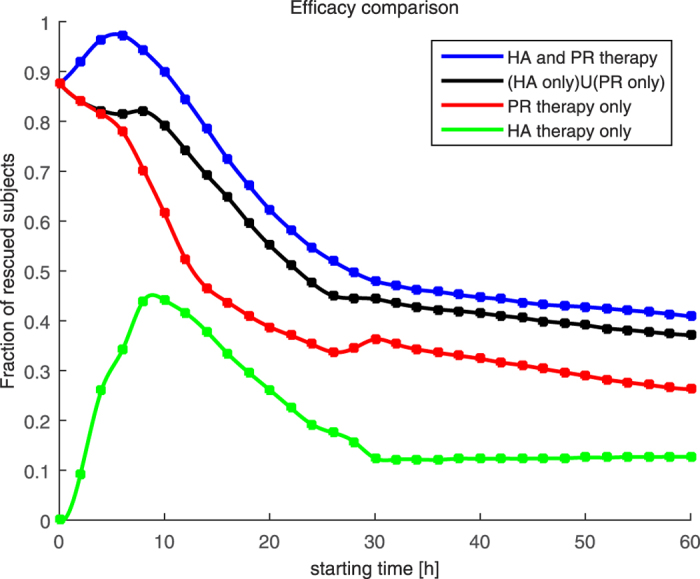
The most effective combination of therapies on 5,000 virtual subjects. The most effective combined therapy is presented by the blue line. Hemoadsorption and pathogen reduction parts of the corresponding combined therapy are depicted with green and red color, respectively. The black line shows accumulated performance of individual therapies. The blue line above the black line provides evidence that there is a synergistic effect when therapies are applied together.

It is shown in Fig. 3 that 24-hour application of the combined therapy has the maximum effect when pathogen reduction therapy is applied longer than hemoadsorption therapy and both of them are applied early. We have plotted a blue squared line that depicts therapies with maximum number of rescued subjects at each starting time. Indeed, we see the pattern that pathogen reduction lasts longer than hemoadsorption therapy. That does not mean that we need to apply only pathogen reduction therapy and ignore hemoadsorption therapy because pathogen reduction is applied for a longer time, but rather that the subject needs both therapies, with more application of pathogen reduction than hemoadsorption. To validate that, we have checked at each time point the duration of each applied therapy. Let us assume that at time point *s* we have applied *p* hours of pathogen reduction therapy and *h* hours of hemoadsorption therapy such that *p* + *h* = 24. Then, we have tested the efficacy of applying only *h* hours of hemoadsorption therapy at time point *s*, and the efficacy of applying only *p* hours of pathogen reduction therapy at the time *s* for all 5000 subjects. These are represented as green and red lines in Fig. 4 for all possible starting time *s* = {0, 2, 4, …, 60}. Moreover, we have plotted the percentage of subjects that can benefit from one of the application of *p* hours of pathogen reduction *or h* hours of hemoadsorption therapy or both. This is represented as the black line in Fig. 4. The black line could be interpreted as if we know in advance whether the subject would benefit from *h* hours of hemoadsorption therapy or *p* hours of pathogen reduction therapy and subsequently the appropriate therapy is given. The blue line represents the combined 24-hour therapy (this is the same as the squared blue line in Fig. 3).

It is clear from Fig. 4 that the combined therapy (blue line) rescued more subjects not only than its individual therapy components (red and green lines) but also than the aggregated performance of its individual therapy components (black line). Meaning that combination of different therapeutic approaches is more than just plain superposition of their effects. In fact it possesses the synergistic trend of having greater performance than the sum of its parts. Such a characteristic is a highly desirable property and could be the piece that contemporary management of sepsis is lacking in order to treat sepsis more successfully.

### Assessing synergistic effects using isobole analysis

**Figure 5:**
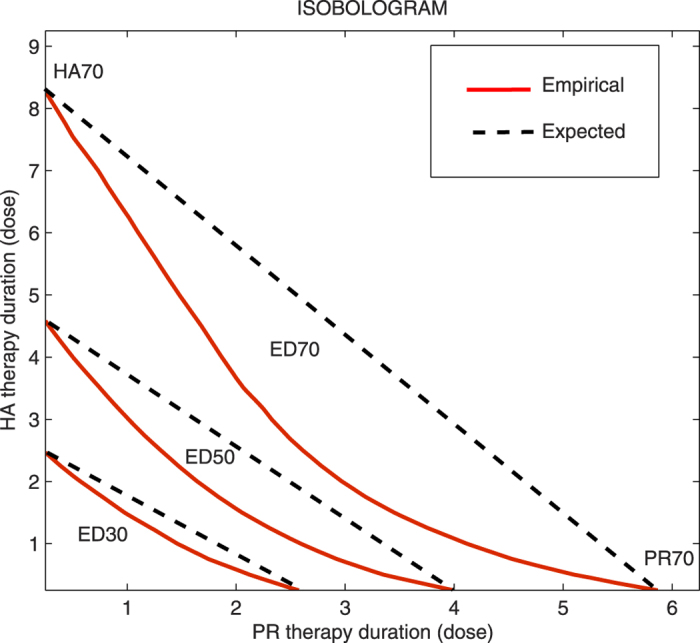
Isobologram of pathogen reduction and hemoadsorption blood cleansing interventions on 5,000 virtual subjects. The isobole (black dotted) lines are representing the expected case of no interaction between the interventions. Empirical lines corresponding to computed lines of targeted efficacy levels for a combined therapy (red lines) show large positive interaction between the components.

The isobole analysis is a well established approach for characterizing the effects of drug combinations in pharmacology studies^38^. Here we will adopt the isobole approach to assess the two blood cleansing interventions. Previously, we have concentrated on survival rate as a variable of interest. However, we have shown its synergistic potential using different reasoning since such a variable isn’t suited for the isobole analysis. That is, if we show that one subject can be saved by either of the therapies, that doesn’t count as two rescued lives. However, the level of pro-inflammatory mediators can be decreased with one intervention, and additionally reduced with the other intervention on top of that. Therefore, we observed the effect of intervention combination on the state of pro-inflammation, one of the crucial states for survival of the subjects. The isobologram in Fig. 5 is derived from individual intervention potencies is shown in SI Appendix Fig. S9.

Commonly, the potency curves are obtained by fitting measured points with a Hill function regression model. However, in this case it was unnecessary, since we were able to calculate curves densely enough using the mathematical model. Obtained curves already visually resemble hyperbolic profiles and attain comparable maximum levels, which are desirable characteristics and result in approximately constant potency ratio between the interventions. The constant potency ratio is assumed in the case where expected combination effects are adopted to be linear, which is used to test the synergism when the interventions are combined. The isobologram in Fig. 5 is derived from individual intervention potencies shown in Fig. S9 of the SI Appendix.

Half of the maximum level of efficiency (ED50) is commonly selected for the isobole analysis, although any level can be chosen. Here we depicted three levels of efficiency, corresponding to 30, 50 and 70% of the maximum (Fig. S9 SI Appendix). The isobole is the line that connects the points with the same expected level of effectiveness in the plane where the axes are the intervention dosages. Commonly the isobole is taken to be the straight line connecting the individual intercepts, which assumes a constant potency ratio^38^. Such approximation fits well in our case given that both individual curves have close maximum effective levels and they both have a hyperbolic profile (SI Appendix Fig. S9), so the linear isobole analysis makes sense. The isobole represents expected effectiveness in the case where no interaction between interventions happens and comparison with the actual measured points will give a clue of possible interaction between the components. If the empirical points are on the isobole line then, as expected, there is no interaction. However, if the points are below the line, there is a synergism. Figure 5 shows that actual computed lines with prescribed levels of effectiveness lies below the derived isobole. These results are indicative of the synergistic interaction happening between the blood cleansing therapies in this model of sepsis, which is yet more evidence that the combination of interventions should be preferable over individual ones.

## Discussion

Presented results provide evidence that blood cleansing therapies could be effective in sepsis management. Earliness and longer therapy application tend to be the most important conditions for successful application. In simulation, typically most of the cases would benefit from both pathogen reduction and hemoadsorption based treatment, but the proposed multiple intervention therapy leads the subject to the recovery state with *maximal* effects and *minimal* efforts. Our extensive simulation modeling of 2,200 configurations of hemoadsorption, pathogen reduction and a combined therapies on 5,000 virtual subjects simulations provides evidence that some subjects could not be treated successfully with a single therapy, while the combination of therapies is beneficial in such cases. In general, a combination of therapies is found more effective as compared to a single approach of the same duration. For an appropriate combination and application time, the 24-fixed-duration combined approach can save almost all virtual subjects (97%). Finally, the combined therapy is more effective as compared to the aggregated performance of its components, and the isobologram analysis is indicative of synergistic interaction of interventions. That synergy between multiple interventions could greatly improve the success of sepsis management.

However, caution should be taken when translating the obtained results to clinical sepsis, because these results were predicted from the mathematical model of rats with CLP induced sepsis. Although the rat CLP sepsis model is considered highly relevant for sepsis in humans^25^, there are still some discrepancies that cannot be neglected. Claiming that the obtained results are relevant for human sepsis based on the rat CLP model is plausible, but indeed might be a distant extrapolation. Specific results, e.g. finding that 21 hours of pathogen reduction with 3 hours of hemoadsorption is the most suitable therapy configuration, cannot be directly translated to humans. However, qualitative claims providing evidence that a combination of therapies is better than a single approach, should be more robust and are likely to generalize well to humans. Even if these claims need additional supporting evidence that they would hold in humans, they are an important step in the process towards drawing conclusions for clinical sepsis. In order to be directly applicable to clinical settings, the model requires some adjustments. The domain knowledge encoded in a mechanistic mathematical model through relations between variables used in this work should still be relevant for the human model, but it would require additional new variables that are considered important in human sepsis, and interrelation thereof. Finally, the model parameters need to be calibrated using relevant data measurements to correspond well to the observations in human sepsis.

Given that in clinical sepsis there are a number of variables that are monitored and stored for evaluating and understanding patient conditions and aid in treatment decisions, they are good candidates for inclusion in the dynamical model of human sepsis. Clinical sepsis is notorious for diversity in manifestation, so rich personal characteristics described by demographics, comorbidities and applied therapies should be able to explain at least a part of the variance. Such an epidemiological dimension of the problem is currently not addressed in animal models of sepsis. In fact, age plays a major role in both incidence and progression of sepsis, and while human septic patients are typically old, animals in sepsis experiments are young and healthy, which might be another source of observational discrepancies. Another important factor in incidence and outcome of clinical sepsis are comorbidities, which often makes sepsis management more difficult. Sex, through hormonal differences, is also found to play a role in the progression of disease. All mentioned characteristics could be incorporated in existing dynamical models that typically account for temporal patterns of concentrations in biomarkers of interest. For example, the effects of age (or sex) could be accounted for by making the parameters of a model a function of such a covariate. In that way, rate of change or maximal concentration of some cytokine can be made to decrease or increase with age. Adding more compartments to the model like the renal or cardiovascular system would allow accounting for comorbidities in some individual by assigning different ranges of parameters, compared to an individual with completely functional systems. Also, effects of applied interventions or administered drugs, like antibiotics and fluid resuscitation, which are considered as a cornerstone of initial management of sepsis^45^, should be incorporated into a human model. Fluid resuscitation effects are commonly modeled so as to affect the cardiac output. Antibiotics could be modeled as an external input that directly affects pathogen state in a similar way that we have modeled pathogen reduction therapy, given that type and doses are appropriate and the main effect of the antibiotic is to kill pathogen cells. One notable difference of effects on the total model would be that pathogen reduction removes both dead and live pathogen cells as well as toxins, while antibiotics do not. Although reducing levels of live bacteria, the remaining dead pathogens and toxins will still be present in the system and continue to attract the attention of the immune system, which will contribute to inflammation. Ideally, the model should be able to account for those effects, either through adding an additional state for dead pathogens, or by directly connecting antibiotics to affect inflammation.

In fact, mathematical models were already extended for applications in the acute inflammation studies in more complex species. The initial model of mice endotoxemia^46^ that incorporated cytokines, their effects on blood pressure, and a general damage term was extended for the lung compartment and adjusted to adapt to a time course of inflammation yielding a porcine model of endotoxemia^47^. Subsequently, a two compartment model of porcine endotoxemia (blood and lungs), was further extended to incorporate another compartment–tissue, and is further adapted to account for human acute inflammation response due to trauma^48^, which was used to characterize the outcome of trauma in terms of levels of IL-6. The rat sepsis model utilized in our work can in a similar way be effectively extended to more complex settings like large animals or clinical sepsis. Also, since the work described here is reanalysis of already existing data, there are various opportunities for experimental verification of conclusions drawn here. As outlined previously, this is only a step towards new therapeutic solutions, and further progress can be made by cyclically repeating model-guided experimental validation and experimentally informed modeling. The result of that process will be knowledge and predictions directly relevant to clinical sepsis, and hopefully a solution to the problem of clinical sepsis treatment.

## Additional Information

**How to cite this article**: Stojkovic, I. *et al.* Effectiveness of Multiple Blood-Cleansing Interventions in Sepsis, Characterized in Rats. *Sci. Rep.* **6**, 24719; doi: 10.1038/srep24719 (2016).

## Supplementary Material

Supplementary Information

## Figures and Tables

**Table 1:** Distribution of 5,000 virtual subjects according the recommended hemoadsorption, pathogen reduction or a combined therapy.

Approach	Hemoadsorption only	Pathogen reduction only	Combination
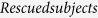	1288	452	3259

Table shows number of subjects and the category of their optimal configuration according to presence of the therapies.
