# Supplementary material for: Effectiveness of Multiple Blood-Cleansing Interventions in Sepsis, Characterized in Rats
Source: Sci Rep. 2016 Apr 21;6:24719. doi: 10.1038/srep24719 (PMC4838820; doi:10.1038/srep24719)
Supplement: Supplementary Information [file srep24719-s1.pdf]

Supplementary Information Appendix

# Effectiveness of Multiple Blood-Cleansing Interventions in Sepsis, Characterized in Rats

Ivan Stojkovic, Mohamed Ghalwash, Xi Hang Cao and Zoran Obradovic

## 1 Materials and Methods

### 1.1 Overall Description of the Sepsis ODE Model

The sepsis mathematical model was proposed for systemic inflammation response (SIR), one of the most significant characteristics of severe sepsis [1]. The model defines the dynamics of concentration for 19 variables (Fig. S1) among which 8 are observable (Lsel - Lselectin; HMGB1 - high mobility group protein B-1; CRT - creatinine; ALT - alanine aminotransferase; TNF$\alpha$ - tumor necrosis factor $\alpha$; IL-1 - interleukin-1$\beta$; IL-6 - interleukin-6; IL-10 - interleukin-10) and 11 are hidden (CLP - cecal ligation and puncture; B - bacteria; Nt - peritoneal neutrophils; Nr - resting blood neutrophils; Np - primed blood neutrophils; Na - activated blood neutrophils; PI - systemic pro-inflammatory response; AI - systemic anti-inflammatory response; Ns - neutrophils sequestered in lung capillaries; Nl - lung neutrophils). The model captures the dynamics of neutrophil trafficking, which responds to the inflammatory stimulus and the interactions between compartments involved during SIR. Those compartments include peritoneum, blood, and lungs. This mathematical model is based on a system of ordinary differential equations (ODEs), whose details are presented bellow.

Sepsis is induced using the cecal ligation and puncture (CLP) procedure. The infection then spreads beyond the infection site, cascades on multiple organ systems, and causes a dysregulated systemic response. In the model, the intensity of the CLP stimulus can be set between 0 and 1, where 0 is the lowest level, and 1 is the highest level in terms of ligation length and puncture size defined by the experimental protocol. Infection is initiated with the sign of logistic growth of bacteria B (Eq. 1). Bacteria grows exponentially upon the start of infection, and slows down as it approaches the capacity of the host. Neutrophils in tissue Nt (Eq. 6) migrate via blood circulation to the infection site to activate phagocytosis.

$$\begin{aligned}\frac{dB}{dt} = s_{CLP}CLP^2 + s_bB(1-\frac{B}{B_{inf}}) - s_NBN_tf_S(B,0,B_{inf}/10)\\ -s_{M0}f_S(B,0,B_{min})\end{aligned} \tag{1}$$

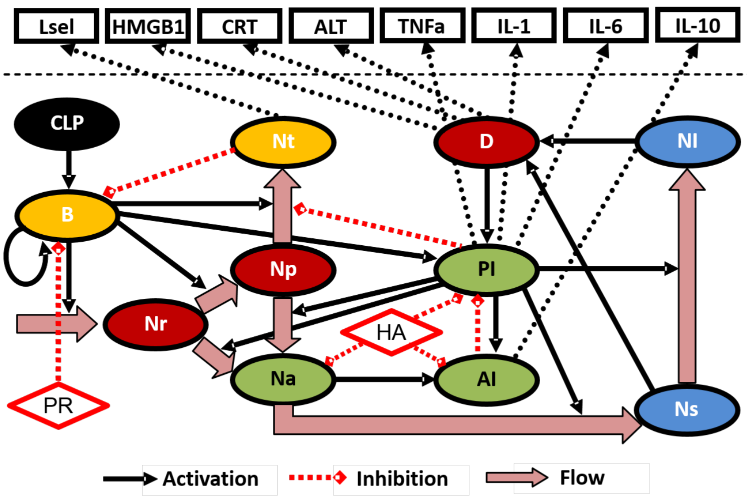


Fig. S1: **The influence network of the mathematical model.** The network representation of 19 states mathematical model [1] depicting the components, interventions and interactions between them. It is generated based on the existing knowledge about the mechanisms underlying the sepsis.

As a reaction to infection, neutrophils are rapidly mobilized from the bone marrow, depicted by the increase of resting neutrophils Nr (Eq. 2) in blood circulation. Resting neutrophils are primed Np (Eq. 3) by interacting with inflammatory mediators, and migrate into the tissue of infection site Nt (Eq. 6). Once the neutrophils become activated, they become sequestered in lung capillaries Ns (Eq. 5). PI (Eq. 8), which stands for pro-inflammatory factors in circulation, inhibits the migration rate of primed neutrophils to the infection site, while it accelerates the activation rate of resting neutrophils. PI can be activated by bacteria B and damage factor D (Eq. 10), but it is inhibited by AI (Anti-inflammatory effect (Eq. 9)).

$$\begin{aligned}\frac{dN_r}{dt} = \frac{1}{\tau_{BM}} + \frac{B}{h_{N_r-B}+B}\frac{1}{\tau_{N_r-B}} - \frac{N_r}{\tau_{N_r}} - \frac{N_r(B/B_{inf})}{h_{N_r-N_p}+(B/B_{inf})}\frac{1}{\tau_{N_r-N_p}} \\ - \frac{N_rPI^n}{h^n_{N_r-N_a}+PI^n}\frac{1}{\tau_{N_r-N_a}}\end{aligned} \tag{2}$$

$$\begin{aligned}\frac{dN_p}{dt} = \frac{N_r(B/B_{inf})}{h_{N_r-N_p}+(B/B_{inf})}\frac{1}{\tau_{N_r-N_p}} - \frac{N_p}{\tau_{N_p}} - \frac{N_pPI^n}{h^n_{N_p-N_a}+PI^n}\frac{1}{\tau_{N_p-N_a}} \\ - \frac{N_p(B/B_{inf})}{h^n_{N_p-N_t}+(B/B_{inf})}\left(1-\frac{PI^n}{h^n_{N_p-N_t}+PI^n}\right)\frac{1}{\tau_{N_p-N_t}}\end{aligned} \tag{3}$$

$$\begin{aligned}\frac{dN_a}{dt} = \frac{N_rPI^n}{h^n_{N_r-N_a}+PI^n}\frac{1}{\tau_{N_r-N_a}} + \frac{N_r(B/B_{inf})}{h_{N_r-N_p}+(B/B_{inf})}\frac{1}{\tau_{N_r-N_p}} \\ - \frac{N_a}{\tau_{N_a}} - \frac{N_aPI^n}{h^n_{N_a-N_s}+PI^n}\frac{1}{\tau_{N_a-N_s}}\end{aligned} \tag{4}$$

$$\frac{dN_s}{dt} = \frac{N_a PI^n}{h^n_{N_a-N_s} + PI^n}\frac{1}{\tau_{N_a-N_s}} - \frac{N_s}{\tau_{N_s}} - \frac{N_s PI^n}{h^n_{N_s-N_l} + PI^n}\frac{1}{\tau_{N_s-N_l}} \tag{5}$$

$$\frac{dN_t}{dt} = \frac{N_p(B/B_{inf})}{h_{N_p-N_t} + (B/B_{inf})}(1 - \frac{PI^n}{h^n_{N_p-N_t} + PI^n})\frac{1}{\tau_{N_p-N_t}} - \frac{N_t}{\tau_{N_t}} \tag{6}$$

$$\frac{dN_l}{dt} = \frac{N_s PI^n}{h^n_{N_s-N_l} + PI^n}\frac{1}{\tau_{N_s-N_l}} - \frac{N_l}{\tau_{N_l}} \tag{7}$$

$$\begin{aligned}\frac{dPI}{dt} = (&\frac{(B/B_{inf})}{h_{PI-B} + (B/B_{inf})}(1 - \frac{D^n}{h^n_{PI-D} + D^n})(1 - \frac{AI^n(1-PI)}{h_{PI-AI} + AI^n}) \\ &+(1 - \frac{(B/B_{inf})}{h_{PI-B} + (B/B_{inf})})\frac{D^n}{h_{PI-D} + D^n}(1 - \frac{AI^n(1-PI)}{h_{PI-AI} + AI^n}) \\ &+\frac{(B/B_{inf})}{h_{PI-B} + (B/B_{inf})}\frac{D^n}{h_{PI-D} + D^n}(1 - \frac{AI^n(1-PI)}{h^n_{PI-AI} + AI^n}) - PI)\frac{1}{\tau_{PI}}\end{aligned} \tag{8}$$

$$\begin{aligned}\frac{dAI}{dt} = (&\frac{PI^n}{h^n_{AI-PI} + PI^n}(1 - \frac{(Na/N_{inf})}{h_{AI-Na} + (Na/N_{inf})}) \\ &+(1 - \frac{PI^n}{h_{AI-PI} + PI^n})\frac{(N_a/N_{inf})}{h_{AI-N_a} + (N_a/N_{inf})} \\ &+\frac{PI^n}{h_{AI-PI} + PI^n}\frac{(N_a/N_{inf})}{h_{AI-N_a} + (N_a/N_{inf})} - AI)\frac{1}{\tau_{AI}}\end{aligned} \tag{9}$$

$$\begin{aligned}\frac{dD}{dt} = (&\frac{(Na/N_{inf})}{h_{D-Na} + (Na/N_{inf})}\frac{(Pp/P_{inf})}{h_{D-Pp} + (Pp/P_{inf})} \\ &+(1 - \frac{(Na/N_{inf})}{h_{D-Na} + (Na/N_{inf})})(1 - \frac{(Pp/P_{inf})}{h_{D-Pp} + (Pp/P_{inf})}) \\ &+\frac{(Na/N_{inf})}{h_{D-Na} + (Na/N_{inf})}(1 - \frac{(Pp/P_{inf})}{h_{D-Pp} + (Pp/P_{inf})}) - D)\frac{1}{\tau_D}\end{aligned} \tag{10}$$

While the internal states (PI, AI, and D) are conceptual and unobservable, the model hypothesized that the plasma cytokines are proxies of that internal states, and their causal

relationships with the internal states are sigmoidal. In particular, HMGB1 (Eq. 15), CRT (Eq. 16) and ALT (Eq. 17) are related with D; TNF (Eq. 11), IL-$1_\beta$ (Eq. 12), and IL-6 (Eq. 13) are related with PI; IL-10 (Eq. 14) is related with AI; Lsel (Eq. 18) is related with the neutrophil amount in the infected tissue.

$$\frac{dTNF}{dt} = (\frac{PI}{h_{TNF} + PI} - TNF)\frac{1}{\tau_{TNF}} \tag{11}$$

$$\frac{dIL1}{dt} = (\frac{PI}{h_{IL1} + PI} - IL1)\frac{1}{\tau_{IL1}} \tag{12}$$

$$\frac{dIL6}{dt} = (\frac{PI}{h_{IL6} + PI} - IL6)\frac{1}{\tau_{IL6}} \tag{13}$$

$$\frac{dIL10}{dt} = (\frac{AI}{h_{IL10} + AI} - IL10)\frac{1}{\tau_{IL10}} \tag{14}$$

$$\frac{dHMGB1}{dt} = (\frac{D}{h_{HMGB1} + D} - HMGB1)\frac{1}{\tau_{HMGB1}} \tag{15}$$

$$\frac{dCRT}{dt} = (\frac{D}{h_{CRT} + D} - CRT)\frac{1}{\tau_{CRT}} \tag{16}$$

$$\frac{dALT}{dt} = (\frac{D}{h_{ALT} + D} - ALT)\frac{1}{\tau_{ALT}} \tag{17}$$

$$\frac{dLsel}{dt} = (\frac{N_a}{h_{Lsel} + N_a} - Lsel)\frac{1}{\tau_{Lsel}} \tag{18}$$

**Virtual Subjects** For the purpose of conducting computational experiments, we have generated 5000 virtual subjects that exhibit the properties of non-surviving animals, using the codes from [1] that can be found at [2]. As shown in Fig. S2, the generated virtual subjects follow similar trajectory of sepsis dynamics as the experimental observations of eight cytokines measured in CLP rats that did not survive one week after the induction of sepsis [1]. The important role of cytokines as bio-markers of sepsis is well acknowledged [3], and the results of the experiments on the virtual subjects should, to some extent, mimic the real-life scenario.

**Hemoadsorption Therapy Model** The model is capable of simulating blood filtration by hemoadsorption (HA) device, which is a column packed with beads that adsorb cytokines to remove the inflammation mediators from circulation. It is assumed that the device, which has two states ON and OFF ($C_{PR}$ is a binary indicator of filtration), is attached to the subject. As long as the device is turned ON, the blood is redirected through the hemoadsorption device, where activated neutrophils Na (Eq. 19) and pro-

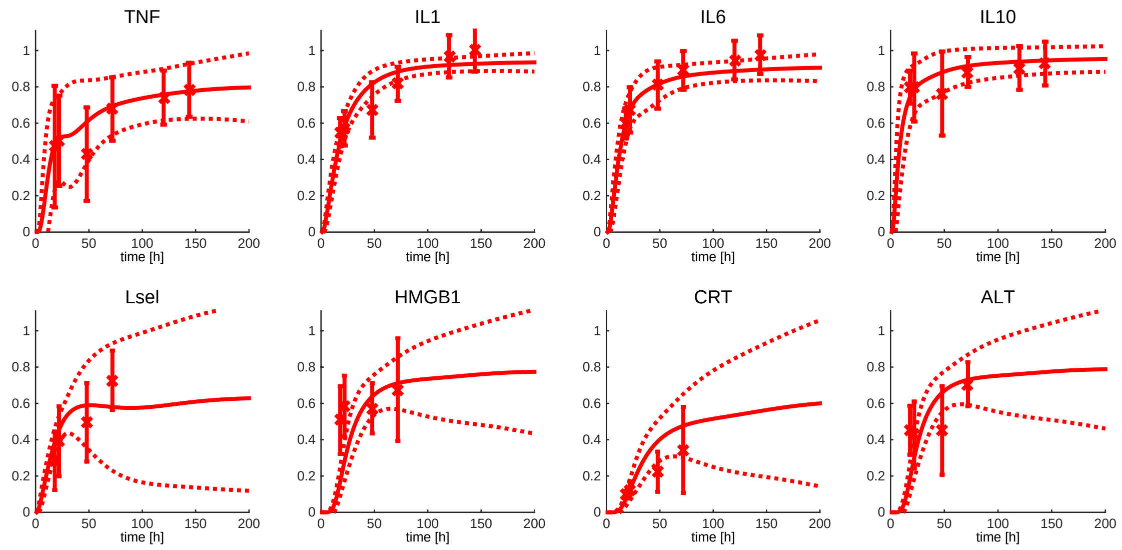


Fig. S2: **An agreement between virtual and real data distribution for 8 cytokines' levels.** Solid lines represent mean values of simulation outputs of 5000 virtual patients in the non-survival group. Dotted lines represent a region of 95% simulation uncertainty (95% of virtual subjects are within this region). Error bars represent real observation statistics from animal study experiments.

(Eq. 20) and anti-inflammatory (Eq. 21) particles are removed, with a rate specified in [1].

$$\frac{dN_{a+HA}}{dt} = \frac{dN}{dt} - C_{HA}(t)\frac{N_a/N_\infty}{h_{NAHA} + N_a/N_\infty} \tag{19}$$

$$\frac{dPI_{+HA}}{dt} = \frac{dPI}{dt} - C_{HA}(t)\frac{PI}{h_{PIHA} + PI} \tag{20}$$

$$\frac{dAI_{+HA}}{dt} = \frac{dAI}{dt} - C_{HA}(t)\frac{AI}{h_{AIHA} + AI} \tag{21}$$

**Pathogen reduction Therapy Model** We have augmented the previously described model to incorporate pathogen reduction (PR) therapy that influences acute inflammation. The effect of PR therapy is simulated by a PR blood filtration device that is attached to the subject. The PR device has two states: ON and OFF. As long as the device is ON the therapy removes the pathogen cells directly. This is simulated as reducing the level of bacteria $B$ (Eq. 22), with a rate of reduction $k_{PR}$ as described by ODE in Eq. (22).

$$\frac{dB_{+PR}}{dt} = \frac{dB}{dt} - k_{PR}C_{PR}(t)B \tag{22}$$

In Eq. (22), $C_{PR}$ represents the state of the device (ON and OFF). The value of the parameter $k_{PR}$ could be estimated as $-ln(0.1)$ (under the convenient assumption of the constant level of pathogen) such that 90% of pathogens are reduced in one hour, as suggested by [4]. Since the pathogen state in the model represents lumped pathogen levels, the part that spillover into blood is just a fraction of that. Therefore, we have adopted a value of the reduction rate parameter to be several times smaller ($k_{PR} = 0.5$). Here we implicitly assumed that both quantities of pathogen have equivalent influence on other variables, thus removing the need to introduce separate states for local and circulating pathogens.

### 1.2 Optimal Combined Therapy

The basic idea is to allow the application of both therapies and let the optimization find out which configuration is the most appropriate. In case it suggests that only one therapy should have nonzero duration, it would mean that single therapy is the best solution. Conversely, if both durations are nonzero (positive), the combined therapy is better. Intuitively, the most appropriate configuration would be the one that leads the subject to a healthy state (inflammation and pathogen levels are below certain thresholds at appropriate time-point) and the lower the pathogen and inflammation levels are, the better the configuration is. Since we have two different therapies HA and PR, we need to identify when and how long to apply each of these therapies. That is, we need to find a discrete control signal that will tell us when the device should be turned ON and when it should be turned OFF. This problem resembles the bang-bang control problem, in which the solution is to find a series of switching times in order to lead the subject towards the healthy state in a way that optimizes the criterion function. More precisely, it is the bang-bang problem with fixed terminal time and free terminal states. However, instead of solving the resulting bang-bang optimal control problem, we have turned it into a finite optimization problem by introducing additional assumptions in the problem. Other than simplifying the problem, an additional reason for that reformulation is that the vast majority of the studied bang-bang problems in literature are free terminal time fixed terminal states problems, i.e. time optimal problems, which is totally opposite of our situation. Moreover, general approaches to bang-bang control problems assume only one external control signal, while we have multiple (two) inputs, which would prevent their direct application. In particular, we assume that we have only one continuous application per therapeutic approach, that is one turning ON and one turning OFF time-point. We will continue to describe this as the configuration of starting time and duration for each therapy.

Given that we have two independent therapeutic approaches, what we need to optimize is the four variables, “starting time” and “duration” for each of the two therapies. The parameters $u$ of the optimization function are given as in Eq. (23).

$$u = [HA_{start}, HA_{duration}, PR_{start}, PR_{duration}]. \tag{23}$$

Then, we need to find a configuration $u$ that minimizes the inflammation level $I$ and the pathogen level $P$. In addition, we prefer to apply the therapy for fewer hours as long as it leads the subject to the healthy state $S$=0, because of the risks associated with

the use of extracorporeal devices [5]. Therefore, the resulting optimization problem is formulated with Eq. (24).

$$\min_{u} w_0 S_t(u) + w_1 P_t(u) + w_2 I_t(u) + w_3 T_t(u) \tag{24a}$$

$$\text{subject to } u(1), u(3) \geq 0 \tag{24b}$$

$$u(1), u(3) \leq 60 \tag{24c}$$

$$T_t(u) = u(2) + u(4) \leq 24h \tag{24d}$$

The objective function in Eq. (24a) takes into account the survival indicator at the terminal time $S_t$, which indicates whether the subject has died (1) or not (0), and associates large penalty if the subject has died. Next, it penalizes the pathogen level $P_t$ and the inflammation level $I_t$ at the termination time, forcing the lower values of those variables. The function also penalizes total duration $T_t$ of the therapy such that the goal is to find the *shortest* effective therapy. The constraints Eq. (24b) and Eq. (24c) are included to bound the search space to a reasonable time-frame, while the constraint Eq. (24d) is added to ensure that the total duration of both therapies is not longer than 24 hours. Since our systems' state at the terminal time is specified by the system dynamics and switching times, we have used numerical integration to obtain its approximation, particularly Runge-Kutta fourth order solving method as implemented in the MATLAB ode45 function.

The resulting nonlinear constrained optimization problem Eq. (24) was solved using the metaheuristic algorithm Cuckoo Search [6] to find a suitable initial point from which the Nelder-Mead simplex algorithm is used to further refine that solution, as provided in the source code [7].

## 2 Supplementary Results

**Connection to Published Work** In previous research on HA therapy [1] authors reported that application of 4 hours of HA therapy starting at the 18th hour after the induction is able to rescue about 18% of non-survival subjects (black point in Fig. S3). In addition, a recent article [8] presented results evaluated for three different durations (4-, 8-, 12-hour therapy applications) at different starting times, as shown in Fig. S3. The results from the literature are consistent with our findings.

Interesting patterns are found from an analysis of 4 hour HA therapy over various starting times (lime colored line in Fig. S3). Application of such therapy in the initial 5 hours (too early) after the induction of sepsis is not as good as application from 5 to 25 hours. These results suggest that silencing inflammation (HA therapy) too early leads to the unobstructed growth of the pathogen, and more pathogen inflicted damage. The interval from 5-25 hours is an appropriate time to start reducing inflammation as it provides the opportunity for inflammation to decrease the pathogen level without causing too much tissue damage. After that period (late application), HA therapy can rescue only subjects in which inflammation is not severe enough to inflict irreparable damage. This is consistent with assumptions that inflammation itself is not a bad but in fact a very essential process, and that only dis-regulated inflammation leads to detrimental effects.

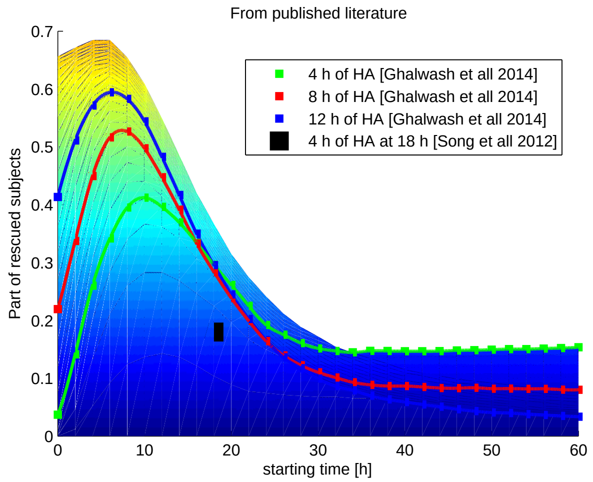


Fig. S3: **Rescue rates of HA therapy reported in published literature.** Compiled results from the published studies of efficacy of HA. Three curves are from [8] and squared dot from [1]. The colored surface represents our results of HA therapy evaluation (from Fig. 1 in the main article) projected on the corresponding plane.

In early stages of infection, an appropriate level of inflammation is necessary, while too little or too much inflammation could lead to undesired effects.

**Success of Therapies and Distribution of Subjects According to Severity** For ease of presentation, we have categorized all subjects into five bins according to the percentage of successful treatments. For example, (0%-10%] indicate the percentage of subjects who could be cured by some particular $x \in (0 - 10]\%$ of configurations (e.g., 10% out of all $31 \times 24 = 744$ possible configurations). In other words, some subjects in that bin could be cured by 1% of configurations, some other subjects could be cured by 2% of configurations, and so on. Therefore, all subjects who could be cured by some $x \in (0 - 10]\%$ of configurations are categorized in the bin (0-10]%. The results for HA therapy are presented in Fig. S4, while a similar graphic for PR therapy is shown in Fig. S5. Percentages of tested therapies that have resulted in rescuing from sepsis can also tell something about the severity of the particular case. In other words, the lower the percentage of successful configurations is, the more severe the case can be considered.

Since for both treatments there exists a group of subjects that cannot be rescued, we have also observed where the distributions of curable and incurable subjects overlap. The resulting four groups are depicted in Fig. S6. It turned out that about one third of the subjects benefit from only one approach, while there also exists a small group of 71 subjects that couldn't be saved with either of the approaches.

**Effect of Optimized Therapies** The distribution of the ten most informative states, pathogen, pro-inflammation levels, and eight cytokine concentrations in subjects without therapy and with prescribed optimal therapy, are depicted in Fig. S7. After the spiking in

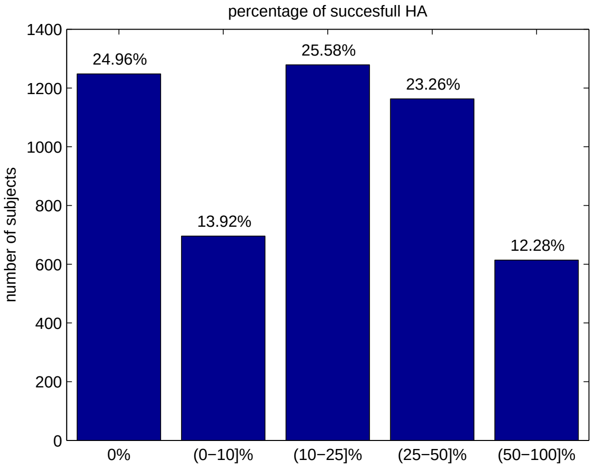


Fig. S4: **Rates of success of HA therapy.** The first bin is the percentage of subjects who could not be cured by any configuration. The last bin is the percentage of subjects who could be cured by most of the configurations (50%-100%]. One in four subjects cannot be cured with HA therapy.

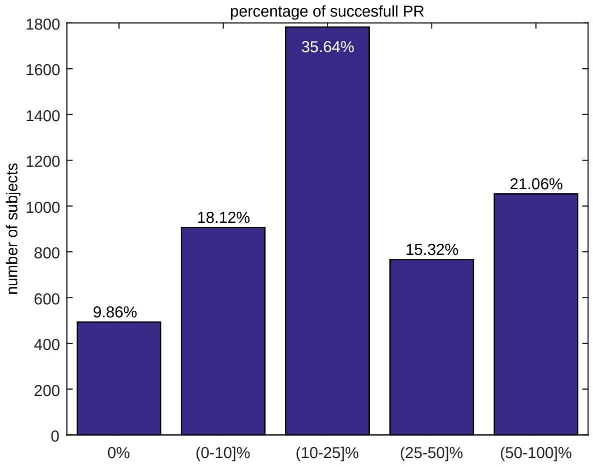


Fig. S5: **Rates of success of PR therapy.** The first bin is the percentage of subjects who could not be cured by any configuration. The last bin is the percentage of subjects who could be cured by most of the configurations (50%-100%]. One in ten subjects cannot be cured with PR therapy.

the states as a reaction to initial stimulus, a steady decrease towards the nominal levels can be observed when the optimal combination of PR and HA therapies is applied. Such a pattern carries the message of resolving the sepsis and returning to the healthy state. Most importantly, values of the pathogen and inflammation levels are decreasing substantially below the threshold for declaring the subject as a non-survivor.

The optimized therapy was able to rescue all but one subject, and combination of therapies was proposed for about two thirds of them. Note that when the optimization

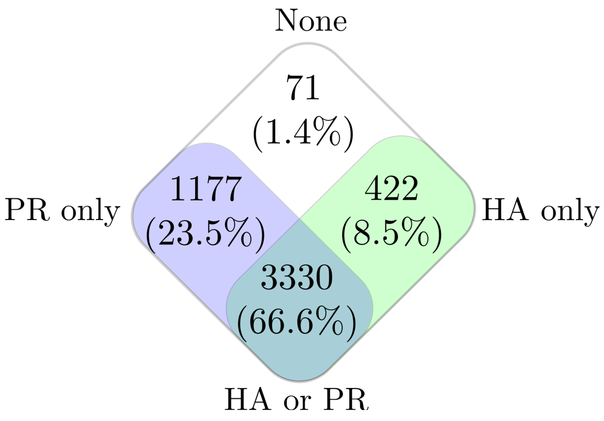


Fig. S6: **Distribution of 5000 subjects based on therapy efficacy.** Purple (green) area represents the number of subjects who could be cured by only PR (HA) therapy, respectively. The overlap between these two sets (dark green) is the number of subjects who could be rescued by either therapy. The white area is the number of subjects who could not be rescued by either of the approaches.

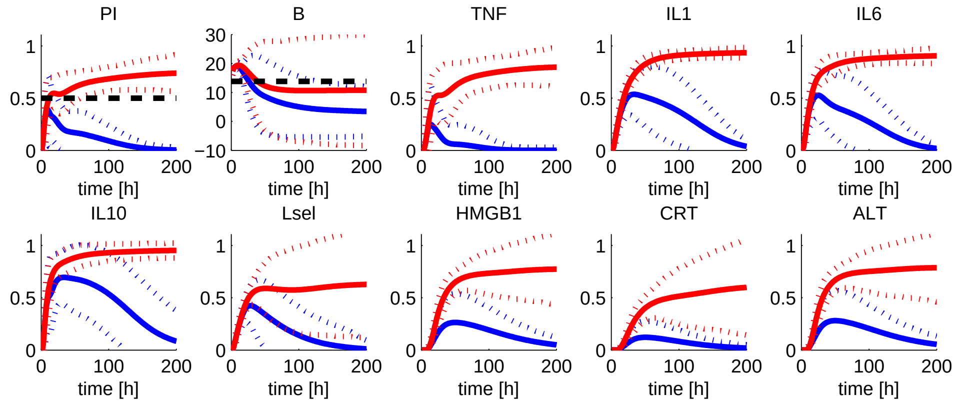


Fig. S7: **Distributions of temporal progression of important states in 5000 virtual subjects without and with optimized therapy.** The solid blue line represents the mean values in simulation of subjects with the prescribed optimal combination of therapies, and the red line represents the mean values of subjects without these therapies. Dotted lines represents borders of 95% confidence levels. The plots for pathogen B and pro-inflammation PI contain the dotted line representing the survival threshold. It can be noticed that with optimal therapy trajectories were moved below the thresholds.

algorithm finds that the optimal configuration would be applying both therapies, it does not mean that the subject could not be saved by either therapy alone. It just means that according to our criteria, the intervention by both therapies minimizes the optimization function more than the intervention by either therapy alone. Nevertheless, the combined approach managed to save 70 out of 71 most severe cases that previously couldn't be saved with either of the approaches alone. We have looked carefully at those 70 subjects who could be saved only by the combination of both therapies. The results are shown in Fig. S8, where we show the cumulative distribution of the total duration of combined therapy application. It can be observed that approximately 60% of those subjects (40 subjects) could be saved by less than 14 hours of combined therapy application, while those subjects could not be saved previously with up to 24 hours of either therapy separately.

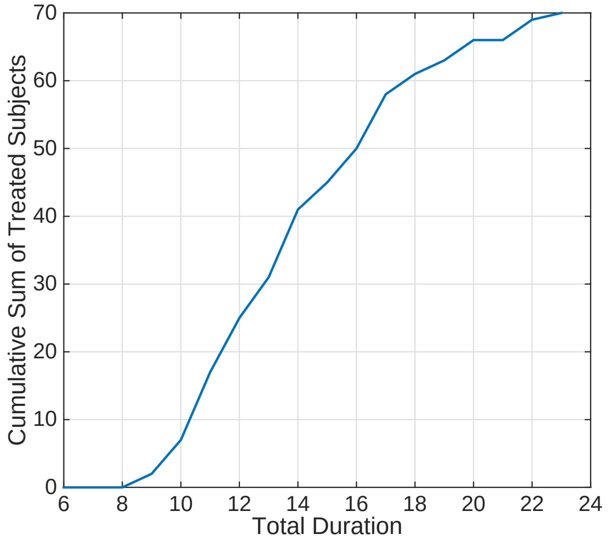


Fig. S8: **Cumulative distribution of subjects cured only by the combined therapy.** The distribution of total application time of combined therapy that leads to recovering from sepsis for those 70 most difficult subjects.

**Isobole analysis** Isobole approach to characterizing the interaction between drugs is based on comparing the expected effects against measured effects of drug combination when part of the dose of one drug is replaced with dose effect equivalent of the other drug [9]. Here we have replaced dose with the duration of blood cleaning, and have fixed the starting time of the intervention at the sixth hour after the induction, since we previously found it as a favorable point for obtaining the maximal effects. Expected effects are found using the individual intervention potency curves as depicted in Fig. S9. The curves are representing the average decrease in pro-inflammatory state relative to the untreated subject's average.

Commonly, the potency curves are obtained by fitting (a few) measured points with a Hill function regression model. However, in this case it was unnecessary, since we were able to calculate curves densely enough using the mathematical model. Obtained curves

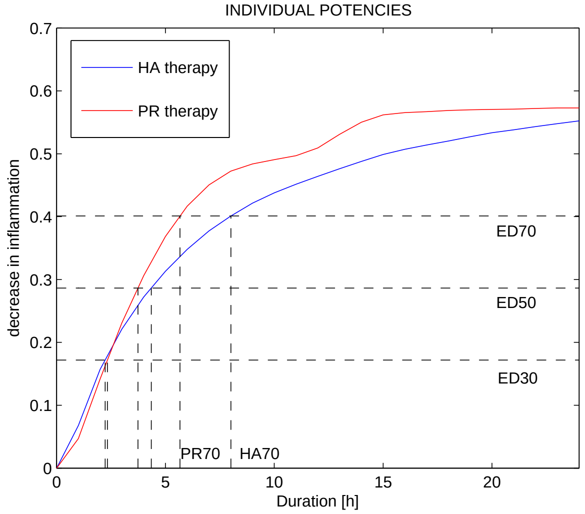


Fig. S9: **Individual interventions' inflammation reducing effectiveness.** The individual ability of two interventions to decrease pro-inflammatory mediator levels are shown as a function of the duration time. The horizontal dotted lines (effective dosage) ED30, ED50 and ED70 are depicting 30, 50 and 70% levels of maximum effectiveness, respectively. The vertical lines represent dosage equivalents of two interventions, that is the duration of each intervention individually needed to achieve desired effect level.

already visually resemble hyperbolic profiles and attain comparable maximum levels, which are desirable characteristics and result in approximately constant potency ratio between the interventions. The constant potency ratio is assumed in the case where combination effects are expected to be linear, which is used in the main article to show synergism when interventions are combined (Fig. 5).

# References


[1] Song SO, Hogg J, Peng ZY, Parker R, Kellum JA, Clermont G. Ensemble models of neutrophil trafficking in severe sepsis. PLoS computational biology. 2012;8(8):e1002422.

[2] Sepsis ODE Code; 2011. `http://code.google.com/p/source-code-sepsis-model/`.

[3] Hack CE, Aarden LA, Thijs LG. Role of Cytokines in Sepsis. Advanced Immunology. 1997;66:95–101.

[4] Kang JH, Super M, Yung CW, Cooper RM, Domansky K, Graveline AR, et al. An extracorporeal blood-cleansing device for sepsis therapy. Nature medicine. 2014;20:1211–1216.

[5] Solberg RG. Extracorporeal circulation: effect of long-term (24-hour) circulation on blood components. Virginia Polytechnic Institute and State University; 2010.

[6] Yang XS, Deb S. Cuckoo search via Lévy flights. In: World Congress on Nature Biologically Inspired Computing. IEEE; 2009. p. 210–214.

[7] Blood Cleansing Code; 2015. `http://www.dabi.temple.edu/~tuf62510/sepsis/blood_cleansing/`.

[8] Ghalwash M, Obradovic Z. A Data-Driven Model for Optimizing Therapy Duration for Septic Patients. In: Proceeding of the 14th SIAM International Conference of Data Mining, 3rd Workshop on Data Mining for Medicine and Healthcare. Philadelphia, PA, USA; 2014.

[9] Tallarida RJ. Drug synergism: its detection and applications. Journal of Pharmacology and Experimental Therapeutics. 2001;298(3):865–872.
